# Expression and localisation of thymosin beta-4 in the developing human early fetal heart

**DOI:** 10.1371/journal.pone.0207248

**Published:** 2018-11-09

**Authors:** Vinay Saunders, Jennifer M. Dewing, Tilman Sanchez-Elsner, David I. Wilson

**Affiliations:** 1 Institute for Developmental Science, Faculty of Medicine, University of Southampton, Southampton, United Kingdom; 2 Academic Unit of Clinical and Experimental Sciences, Faculty of Medicine, University of Southampton, Southampton, United Kingdom; IRCCS San Raffaele Pisana, ITALY

## Abstract

**Background:**

The objective of this study was to investigate the expression and localisation of thymosin β4 (Tβ4) in the developing human heart. Tβ4 is a cardioprotective protein which may have therapeutic potential. While Tβ4 is an endogenously produced protein with known importance during development, its role within the developing human heart is not fully understood. Elucidating the localisation of Tβ4 within the developing heart will help in understanding its role during cardiac development and is crucial for understanding its potential for cardioprotection and repair in the adult heart.

**Methods:**

Expression of Tβ4 mRNA in the early fetal human heart was assessed by PCR using both ventricular and atrial tissue. Fluorescence immunohistochemistry was used to assess the localisation of Tβ4 in sections of early fetal human heart. Co-staining with CD31, an endothelial cell marker, and with myosin heavy chain, a cardiomyocyte marker, was used to determine whether Tβ4 is localised to these cell types within the early fetal human heart.

**Results:**

Tβ4 mRNA was found to be expressed in both the atria and the ventricles of the early fetal human heart. Tβ4 protein was found to be primarily localised to CD31-expressing endothelial cells and the endocardium as well as being present in the epicardium. Tβ4-associated fluorescence was greater in the compact layer of the myocardial wall and the interventricular septum than in the trabecular layer of the myocardium.

**Conclusions:**

The data presented illustrates expression of Tβ4 in the developing human heart and demonstrates for the first time that Tβ4 in the human heart is primarily localised to endothelial cells of the cardiac microvasculature and coronary vessels as-well as to the endothelial-like cells of the endocardium and to the epicardium.

## Introduction

Understanding the development of the human heart can lead to insights into both congenital and acquired heart disease. The processes that occur during embryonic development of the heart are becoming better understood and a number of molecular factors are now known to play important roles in normal cardiac development [1].

If we understand the roles of such biological molecules in healthy cardiac development this may lead not only to insights into poorly understood congenital heart conditions but may also lead to novel ways to treat and manage acquired cardiac diseases.

Thymosin β4 (Tβ4) is an endogenous protein which was originally identified as an actin sequestering molecule. The known functions of Tβ4 have since expanded to include roles in cell migration [2–4], angiogenesis [5], wound healing [3, 4, 6], cell survival [7–9] and suppression of inflammation [10–12].

These roles have led to interest in the therapeutic potential of Tβ4, particularly in relation to cardiovascular disease due to its reported cardioprotective effects. Exogenous treatment with recombinant Tβ4 leads to reduced infarct sizes, reduced fibrotic scarring and thus improved cardiac output in various *in vivo* models of myocardial infarction [7, 13, 14].

While there is understandable interest in using recombinant Tβ4 therapeutically, Tβ4 is in fact endogenously expressed within the heart and has been shown to have a crucial role in cardiac development [7, 15, 16]. Indeed, using short hairpin RNA to knockdown Tβ4 protein in mice leads to heart defects in embryos, with the greatest levels of Tβ4 knockdown leading to death and resorbtion of the embryo [16].

Thus, Tβ4 has clear importance in normal cardiac development. However, despite this, the endogenous expression and localisation of Tβ4 in the heart is not yet fully defined and while it has been partially described in relation to the murine heart there is a lack of human data.

Within mice, cardiomyocytes have been implicated in the production of Tβ4. Cardiomyocyte-specific knockdown of Tβ4 has been shown to prevent endothelial and smooth muscle cells derived from the epicardium from migrating into the heart, thus preventing coronary vessel formation [16]. This suggests that Tβ4 is produced by cardiomyocytes during early cardiac development and acts upon the epicardium in a paracrine fashion. Indeed, *in situ* hybridisation data presented in the same study demonstrates that Tβ4 mRNA is not present in the epicardium at E14.5, indicating epicardial cells do not themselves produce Tβ4 [16].

Further to this, the presence of Tβ4 mRNA in the ventricles of the murine heart has been demonstrated, with the compact layer of the myocardium and interventricular septum being enriched for Tβ4 mRNA [7, 16]. However, the cell types which express Tβ4 mRNA were not identified in these studies [7, 16].

There is little published data in relation to the localisation of Tβ4 protein within the heart in either mouse or human. In a study by Smart et al, murine heart sections (E14.5) double-stained for detection of Tβ4 and vascular endothelial growth factor receptor 2 showed relatively homogeneous staining of Tβ4 that was absent in patches where vascular endothelial growth factor receptor 2 was present [16]. This led the authors to conclude that Tβ4 protein is not localised to endothelial cells, but is present in other cells of the ventricular myocardium [16]. This complemented the Tβ4 knockdown data shown in their study, suggesting that while Tβ4 is important in endothelial cells development, it is produced by cardiomyocytes. However, in direct contrast to this, data from Banerjee et al shows Tβ4 staining in sections of an E13.5 murine heart in which Tβ4 does appear to be present in the coronary vasculature at higher levels than surrounding myocardium, though the authors themselves do not comment on this finding [17]. In human, a tissue microarray has been used to simply demonstrate that Tβ4 protein is present in the adult human heart [15].

Beyond the heart, Tβ4 mRNA is reported to be present throughout the vasculature of the developing mouse [18]. Additionally, immunofluorescence data illustrates the localisation of Tβ4 protein to endothelial cells in the developing aorta (E10.5), as well as in the smooth muscle wall of the aorta later in development (E12.5) [18]. Studies suggest that Tβ4 plays an important role in vessel formation and may even be involved in blood vessel stability [5, 16, 18].

However, while there is good evidence of an important role for Tβ4 in development of the vasculature both within the heart and throughout the body, some studies have sought to contradict these findings, concluding that Tβ4 is dispensable for murine development [17, 19]. The difference in findings between groups is likely due to differences in the techniques used to explore the functional role of Tβ4. While Smart et al used shRNAs to knock-down Tβ4, Banerjee et al used gene recombination to create knockout mice [16–19]. It is possible that in Tβ4 knockout mice, compensatory effects come in to play during development, with other members of the thymosin family fulfilling the role of Tβ4 and preventing the manifestation of any abnormal phenotype. Such compensatory mechanisms may not come in to play when shRNAs were used, since the knock-down achieved was not 100% and thus Tβ4 itself was still present during embryonic development [16].

Knowledge of the cells types in which Tβ4 is localised within the human heart would allow for a better understanding of how Tβ4 is involved in normal cardiac vascular development as well as how it endogenously mediates cardiac repair; aspects which may be utilised in the clinic. This study demonstrates the expression of Tβ4 in the developing human heart and further describes the localisation of Tβ4 protein.

## Methods

### Human fetal tissue

Human embryonic/fetal material was obtained with informed written consent and full ethical approval (Southampton and West Hampshire Local Research Ethics Committee, 296/00) from women undergoing termination of pregnancy in Southampton. Human embryonic/fetal material was also provided by the Joint MRC/Wellcome Trust (grant #099175/2/12/2) Human Developmental Biology Resource (www.hdbr.org). Cardiac tissue used was from fetuses aged 7–12 post-conception weeks (pcw).

### RNA extraction, reverse transcription and PCR

Total RNA was extracted from cardiac tissue using standard guanidinium thiocyanate-phenol-chloroform RNA extraction methods.

RNA samples were treated with RQ1 RNase-free DNase (Promega), according to the manufacturer’s instructions. Samples were then reverse transcribed to cDNA using M-MLV Reverse Transcriptase (Promega) with Random Primers (Promega), according to the manufacturer’s instructions. ‘No reverse transcriptase’ controls were created for each RNA sample by using water in place of the reverse transcriptase enzyme.

PCR was carried out according to standard protocols using GoTaq polymerase (Promega) in combination with dNTPs and specifically designed primers. Primers were designed using PrimerSelect (Lasergene 8; DNASTAR Inc.). The primer sequences used to detect *TMSB4X* transcripts were as follows.

Forward primer: GAAGACAGAGACGCAAGAGAAAAA

Reverse primer: TGCCAGCCAGATAGATAGACAGAT.

PCR was carried out using a G-Storm GS1 thermocycler, using an annealing temperature of 58°C. The PCR products were resolved on 2% agarose gels containing Nancy-520 (Sigma Aldrich). Gels were imaged using a High Performance Ultraviolet Transilluminator (UVP) and the associated DocIT software (UVP).

DOI: dx.doi.org/10.17504/protocols.io.rvbd62n

### Immunohistochemistry

Fetal hearts were PFA-fixed, embedded in paraffin, cut into 10 μm thick sections and the sections mounted onto aminoalkylsilane-coated glass slides.

Sections were stained according to standard methods using specific antibodies for detection of Tβ4, myosin heavy chain (MHC), CD31 and alpha smooth muscle actin (SMA).

Prior to staining, antigen retrieval was carried out using boiling 0.01 mol/L sodium citrate solution (pH 6.0) + 0.05% Tween 20.

Sections were stained with the appropriate primary and secondary antibodies. Sections stained with secondary antibodies alone were used as negative controls.

The primary antibodies used were as follows: rabbit anti-thymosin β4 polyclonal antibody used at 1:1500 dilution (Millipore Cat# AB6019, RRID:AB_10806893); mouse anti-myosin heavy chain monoclonal antibody used at 1:50 dilution (Thermo Fisher Scientific Cat# 50-6503-80, RRID:AB_2574266); mouse anti-SMA monoclonal antibody used at 1:100 dilution (Leica Microsystems Cat# NCL-SMA, RRID:AB_442134); sheep anti-CD31 polyclonal antibody used at 1:2000 dilution (R and D Systems Cat# AF806, RRID:AB_355617).

The secondary antibodies used were as follows: goat anti-rabbit IgG-FITC at 1:200 dilution (Sigma-Aldrich Cat# F6005, RRID:AB_259682); goat anti-mouse IgG-Alexa Fluor 594 at 1:200 dilution (Thermo Fisher Scientific Cat# A-11005, RRID:AB_2534073); donkey anti-sheep IgG-Alexa Fluor 555 at 1:400 dilution (Thermo Fisher Scientific Cat# A21436, RRID:AB_10376163).

Following staining, the sections were mounted and the nuclei counterstained using Vectashield Mounting Medium with DAPI (Vector Laboratories). Slides were imaged using a standard fluorescence microscope or a Leica SP5 confocal microscope.

DOI: dx.doi.org/10.17504/protocols.io.rwcd7aw

## Results

### Thymosin β4 expression in the developing heart

**Fig 1:**
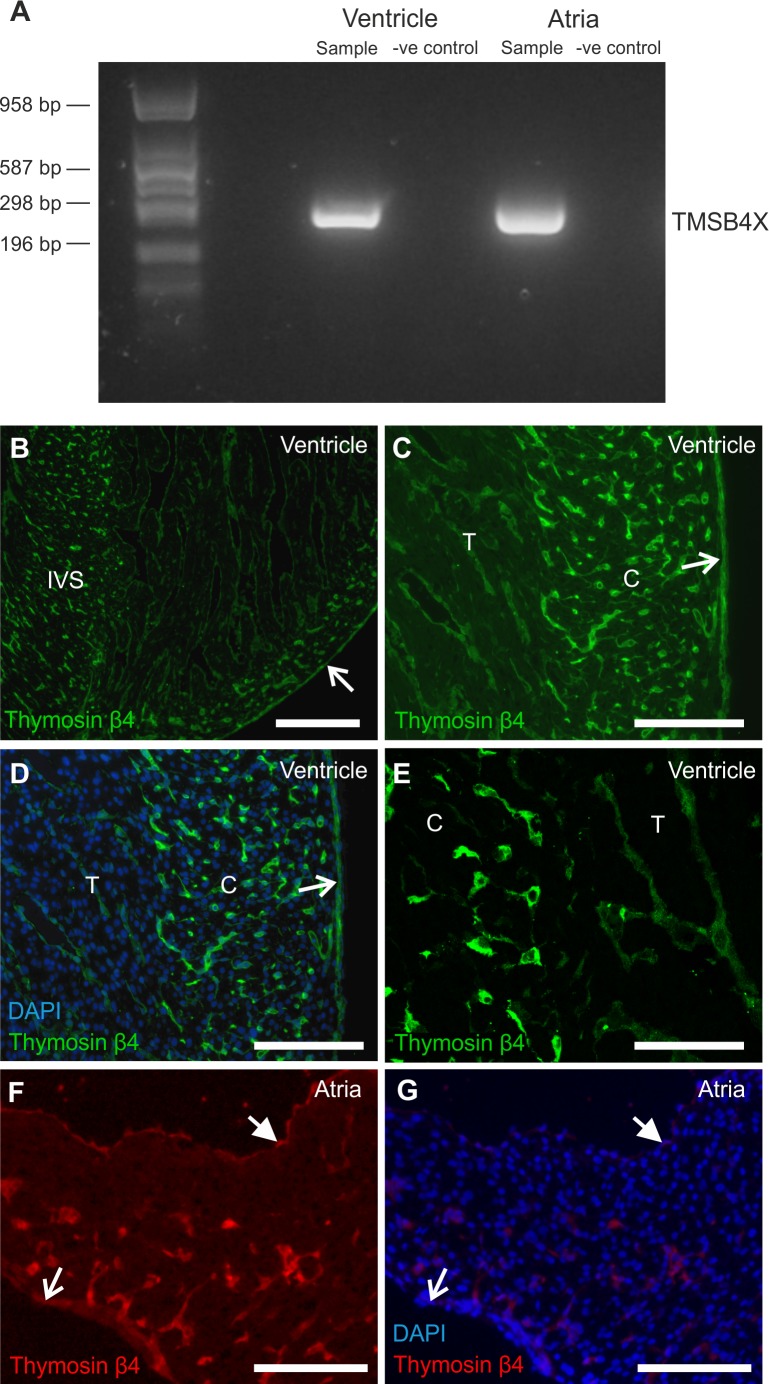
Thymosin-β4 is expressed in the developing human heart. A) Detection of TMSB4X cDNA (278 bp) by PCR in human fetal atria and ventricle samples. Alongside each cDNA sample a corresponding 'no reverse transcriptase' negative control sample (-ve control) was run. Representative of three individual hearts (7–8 pcw). B-G) Fluorescent immunostaining for Tβ4 protein indicates localisation to specific cells in both the ventricle (B, C, D, E) and atria (F & G), as well as to the epicardium (open arrows) and endocardium (closed arrows). DAPI nuclear staining is also shown (D & G). Within the ventricle walls, staining was more intense in the interventricular septum (IVS) and compact layer (C), compared to the trabecular layer (T). Representative of six individual hearts (7–12 pcw). Scale bars = 200 μm (B), 150 μm (C, D, F, G), 100 μm (E).

To confirm that *TMSB4X*, the gene for Tβ4, is expressed in the developing human heart, PCR was carried out using a set of primers designed to amplify any of the four isoforms of *TMSB4X* transcript, without distinguishing between them. PCRs were carried out on cDNA extracted from ventricular and atrial tissue samples from fetal hearts aged 7–8 pcw (n = 3). Bands indicating detection of *TMSB4X* transcripts were seen in both the ventricular and atrial tissue samples (Fig 1A).

With *TMSB4X* expression confirmed, fluorescence immunohistochemistry was utilised to assess the localisation of Tβ4 protein within the human heart. Sections taken from human fetal hearts aged between 7 and 12 pcw were stained for Tβ4 detection (n = 6).

Tβ4 staining was seen in the walls of the ventricles (Fig 1B & 1C) and atria (Fig 1D & 1E). The staining patterns indicated that Tβ4 protein was localised to specific cell types within the walls of both the ventricle and atria and was not expressed homogeneously throughout the myocardial walls.

Within the ventricles, Tβ4 staining was more intense in cells within the compact layer of the myocardium and the interventricular septum compared to the trabecular layer (Fig 1B & 1C). Additionally, Tβ4 was clearly present within the cells of the epicardium (open arrows) and the endocardium (closed arrows) (Fig 1B).

### Thymosin β4 localisation in the ventricle wall

As cardiomyocytes represent the bulk of the volume in the cardiac walls, Tβ4 and MHC dual stains were carried out in order to assess whether Tβ4 was present in cardiomyocytes. MHC is a subunit of the myosin II motor protein and in the heart is a marker of cardiomyocytes.

**Fig 2:**
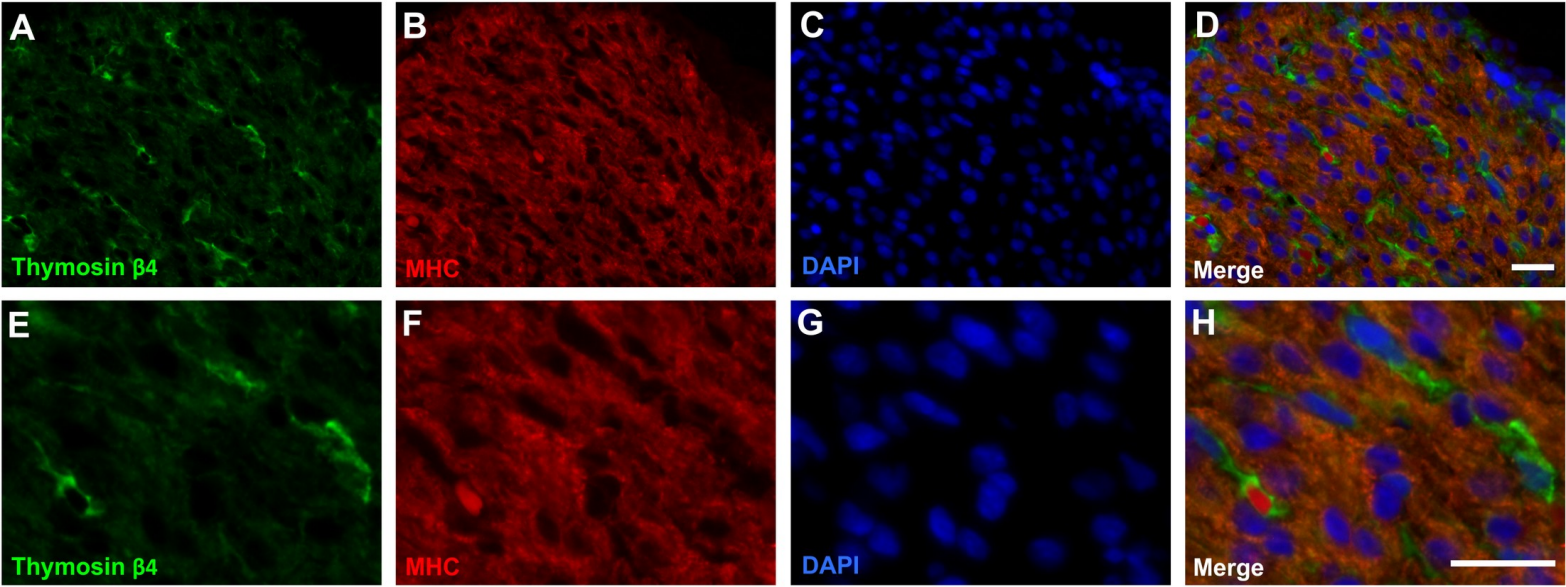
Thymosin-β4 is not co-localised with MHC in the ventricle wall. Fluorescent immunostaining of foetal heart sections dual stained for thymosin β4 (A & E) and MHC (B & F), a cardiomyocyte marker, at low (A-D) and high (E-H) magnification. DAPI nuclear staining is also shown (C & G). Thymosin β4 staining is distinct from MHC staining (H). Representative of six individual hearts (7–12 pcw). Scale bars = 25 μm.

The immunohistochemistry demonstrated that the staining pattern of Tβ4 clearly differed from that of MHC (Fig 2), with no co-localisation seen between the two antibodies (Fig 2D–2H).

**Fig 3:**
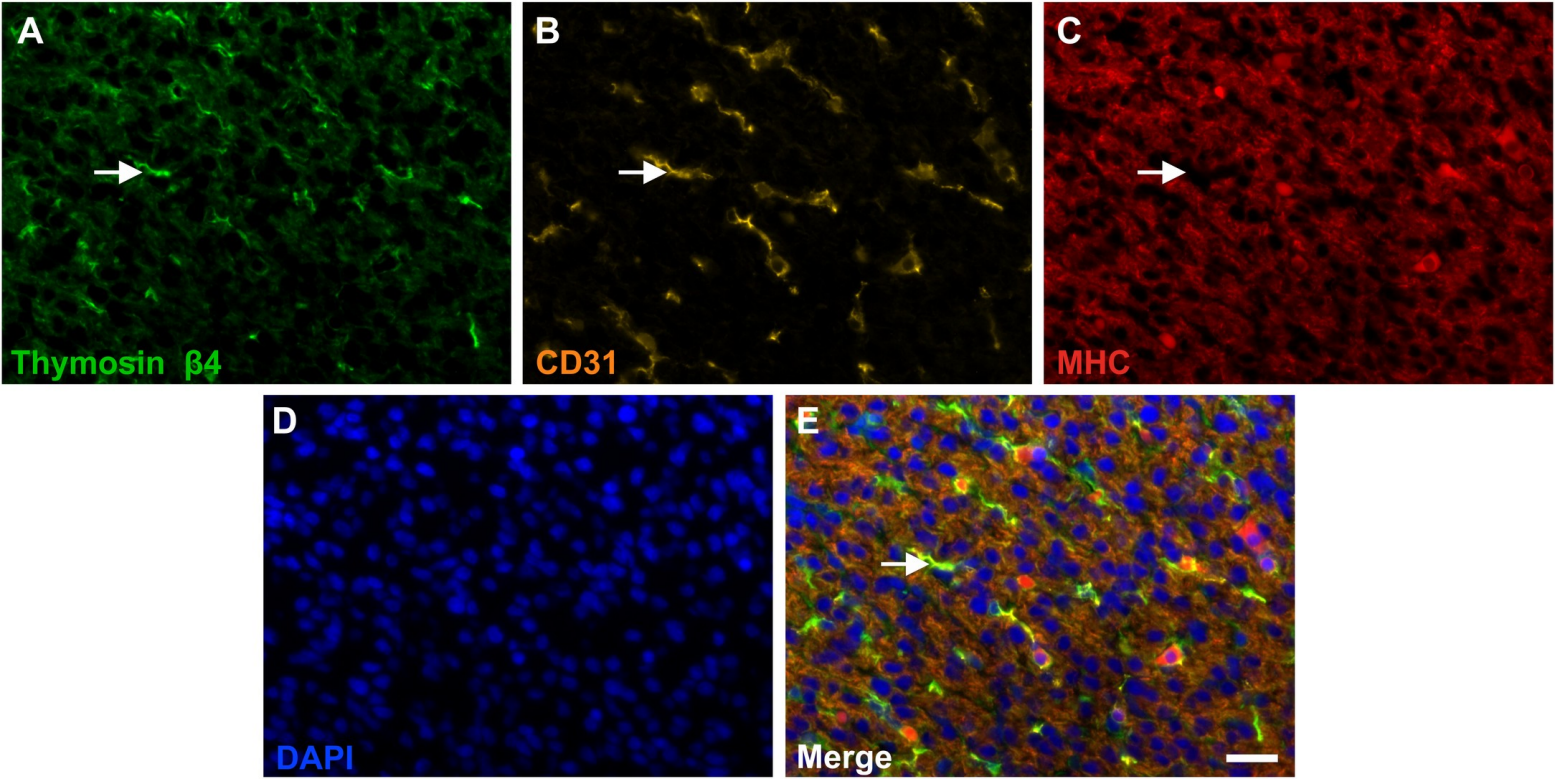
Thymosin-β4 is primarily co-localised with CD31 in the ventricle wall. Triple fluorescent immunostaining of fetal ventricle sections for Tβ4 (A), CD31 (B), an endothelial cell marker, and MHC (C). DAPI nuclear counterstain is also shown (D). The staining pattern of Tβ4 is similar to that of CD31, with large areas of co-localisation (E). Neither Tβ4 nor CD31 co-localises with MHC (closed arrows). Representative of six individual hearts (7–12 pcw). Scale bar = 40 μm.

In order to assess whether Tβ4 was present within endothelial cells, triple staining for detection of Tβ4 alongside CD31, an endothelial marker, as well as MHC was carried out. Areas of Tβ4 staining were clearly co-localised with CD31 staining, indicating the presence of Tβ4 protein in endothelial cells (Fig 3). These Tβ4-positive, CD31-positive areas were absent of MHC staining (Fig 3, closed arrows).

### Thymosin β4 localisation in coronary vessels

**Fig 4:**
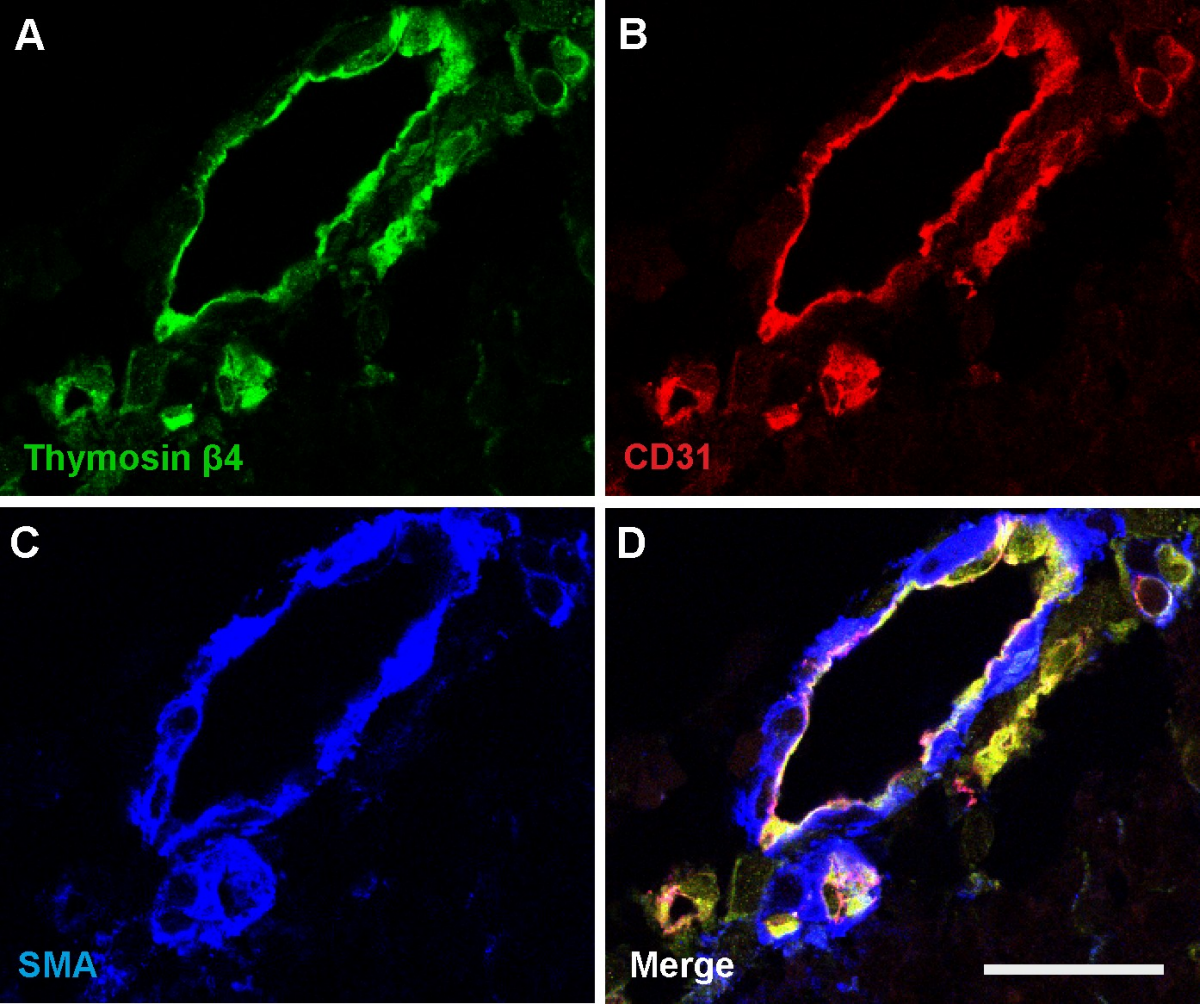
Thymosin-β4 is localised to endothelial cells in the coronary vessels. Confocal imaging of triple fluorescent immunostaining of a section of medium coronary vessel within the ventricle wall. Staining is for Tβ4, CD31 and SMA, a smooth muscle cell marker. The staining pattern indicates that Tβ4 is localised to the CD31-positive endothelial cells, not the smooth muscle cells. Representative of six individual hearts (7–12 pcw). Scale = 25 μm.

As staining indicated the localisation of Tβ4 to endothelial cells in the capillaries of the ventricle wall, the presence of Tβ4 in endothelial cells of coronary vessels was assessed. Triple staining of sections was carried out for detection of Tβ4, CD31 and SMA, a smooth muscle cell maker. The staining indicated that in coronary vessels Tβ4 was co-localised to CD31-positive endothelial cells and not to the smooth muscle cells (Fig 4).

## Discussion

This study has demonstrated the expression of Tβ4 in the developing human heart and illustrated its localisation at the protein level. Tβ4 mRNA was detected in the atria and the ventricles of the developing heart and the presence of Tβ4 protein in both of these structures was confirmed by fluorescence immunohistochemistry.

Further to this, Tβ4 protein was found to be primarily localised to endothelial cells throughout the heart, including in the medium coronary vessels. Tβ4 protein was also present in the endothelial-like cells of the endocardium, and was also detected within cells of the epicardium. The intensity of Tβ4 staining was greater in cells within the compact layer of the heart and the interventricular septum than within the trabecular layer of the heart, indicating these cells express higher levels of Tβ4 protein. Tβ4 staining was not apparent in cardiomyocytes.

Where observations have previously been reported in relation to the murine heart our data largely supports them. While any discrepancies may be explained by genuine differences between species, there are alternative potential explanations in all cases.

In this study Tβ4 gene expression was detected in both the ventricles and the atria using PCR. While expression in the ventricles is consistent with previously reported in situ hybridisation data in the mouse, Bock-Marquette et al [7] did not detect Tβ4 mRNA in the atria. This discrepancy may be explained by a lack of sensitivity using in situ hybridisation compared to PCR or because the cDNA probe used by Bock-Marquette et al may not detect all isoforms of murine Tβ4 transcript. The Ensembl database lists three different isoforms of murine Tβ4 transcript. The cDNA probe used by Bock-Marquette et al is described as binding to the 3’UTR of murine Tβ4 cDNA; however, isoform Tmsb4x-003 is indicated to have a shorter 3’UTR than the other 2 isoforms. This means the probes used for in situ hybridisation may not detect all isoforms, whereas the primers used in the current study were designed to detect any of the 4 isoforms of human Tβ4 mRNA listed in the Ensembl database.

An alternative explanation is that Tβ4 may only be expressed in cells of the atria in the later stages of embryonic development; in the study by Bock-Marquette et al [7], Tβ4 expression was assessed in a murine heart at E12.5, which is the equivalent to Carnegie stage 18/19 in human, while the current study used hearts ranging from Carnegie stage 20 into early fetal development (up to 12 pcw).

There is little previously published data reporting the localisation of Tβ4 protein in the developing heart. In the mouse there are conflicting reports. The data shown here is in distinct contrast to the Tβ4 staining demonstrated by Smart et al [16] in the murine heart which suggests Tβ4 is present homogenously throughout the ventricle wall, but is absent from endothelial cells. In contrast, we show differential expression of Tβ4 between the compact layer and the trabecular layer and illustrate that Tβ4 is most abundantly present within endothelial cells. This differential expression between the compact and trabecular layers of the heart is in fact in agreement with in situ hybridisation data presented not only by Smart et al themselves but also by Bock-Marquette et al [7].

In addition to this, the immunostaining of Tβ4 in endothelial cells but not in cardiomyocytes in this study appears to be consistent with immunofluorescence data presented by Banerjee et al, in which Tβ4 staining is seen in specific cells in the ventricle wall including in structures which appear to be blood vessels, though the authors themselves do not draw this conclusion and simply use the staining to illustrate the presence of Tβ4 protein [17]. The presence of Tβ4 in the endothelial cells of the medium coronary vessels, shown in this study, is also consistent with the observation in mice that Tβ4 is present in blood vessels throughout the body [18].

The differential expression of Tβ4 between the compact and trabecular layers is not unique and similar differential expression is seen with several other genes during cardiac development [20]. The differential expression of Tβ4 specifically may be due to the different developmental origins of the endothelial-like endocardial cells in the trabecular layer compared to the endothelial cells of the coronary vasculature found in the compact layer. The coronary vasculature is derived from the epicardium and migrates into the ventricle wall during formation of the compact layer at a much later stage in development than that at which endocardial cells migrate into the heart [21].

Tβ4 protein was also identified in the epicardium in our study, which may be considered to be consistent with Tβ4’s presence in coronary endothelial cells shown here and with its role in formation of the coronary vessels from the epicardium, shown by Smart et al [16]. However, Tβ4 transcript was not seen in the epicardium of the murine heart at an equivalent stage of development. The cDNA probe used by Smart et al for in situ hybridisation, like that used by Bock-Marquette et al [7], is targeted to the 3’UTR of the murine Tβ4 transcript. Therefore, it may be that an isoform of Tβ4 that is not detected by the probe was present, while, in contrast, human Tβ4 transcripts all produce the same protein, meaning the antibody used here to detect Tβ4 protein would detect protein translated from all isoforms.

While, in the current study, Tβ4 protein was found to be primarily associated with endothelial cells, it is clear from knockdown studies in mice that Tβ4 is produced by cardiomyocytes, at least in the earliest stages of cardiac development, where it is of vital importance [16]. It may be that expression of Tβ4 in the heart changes throughout development, with expression initially in cardiomyocytes but with this expression changing once the epicardium has been activated and the coronary vessels and compact layer begin to form. This hypothesis may be tested by assessing Tβ4 expression within the heart throughout development, something which may be achieved most easily by studying the murine heart.

In conclusion, we show that Tβ4 is expressed in the developing human heart, where it is primarily localised to endothelial cells and cells of the endocardium and epicardium.

Information on the cellular localisation of Tβ4 will be important for the development of therapeutic approaches that utilise the beneficial effects of Tβ4. Therapeutic possibilities include the use of gene therapy to enhance Tβ4 production in relevant cells in a targeted way following a myocardial infarction. Cell therapies also have potential in cardiovascular medicine and, indeed, transplant of modified embryonic stem cells which over-express Tβ4 have been shown to have benefit in a rat model of myocardial infarction [22]. Additionally, Tβ4 has been used to facilitate the production of cardiac patches designed to replace damaged tissue following myocardial infarction [23]. Knowledge of which cells Tβ4 is important to during normal development will help to refine and enhance such cell-based therapies for clinical use.

The finding presented here reinforce the importance of Tβ4 in the development of the vasculature within the heart and may help guide future studies looking to dissect the role of Tβ4 in the developing and mature heart and its potential therapeutic roles in repairing the failing heart.

## Supporting information

S1 Fig Original image of DNA gel shown in Fig 1. (TIF)

S2 Fig Control staining for thymosin β4 antibody. Fluorescent immunostaining images of sections stained for detection of thymosin β4 (A & D), sections probed with the primary anti-thymosin β4 antibody after blocking by pre-incubation with synthetic thymosin β4 (B & E) and sections incubated with the secondary antibody alone (C & F). The distinct staining pattern seen in sections stained for detection of thymosin β4 using the anti-thymosin β4 primary antibody alongside a secondary antibody is not seen in either negative control. Scale bars = 200 μm.
(TIF)
