# Supplementary figures and images for: Expression and localisation of thymosin beta-4 in the developing human early fetal heart
Source: PLoS One. 2018 Nov 9;13(11):e0207248. doi: 10.1371/journal.pone.0207248 (PMC6226193; doi:10.1371/journal.pone.0207248)

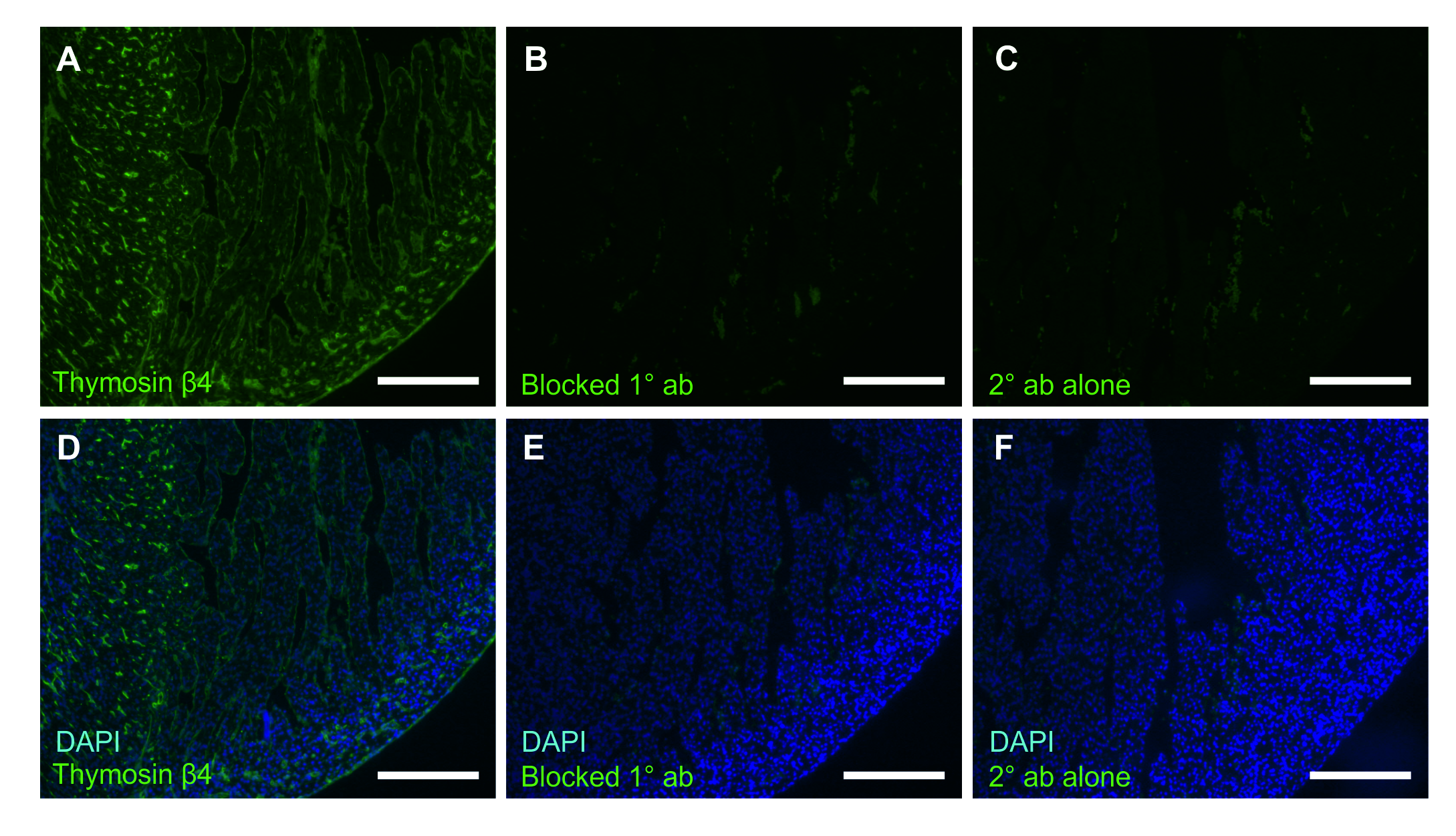

Supplement: S2 Fig — Fluorescent immunostaining images of sections stained for detection of thymosin β4 (A & D), sections probed with the primary anti-thymosin β4 antibody after blocking by pre-incubation with synthetic thymosin β4 (B & E) and sections incubated with the secondary antibody alone (C & F). The distinct staining pattern seen in sections stained for detection of thymosin β4 using the anti-thymosin β4 primary antibody alongside a secondary antibody is not seen in either negative control. Scale bars = 200 μm. (TIF) [file pone.0207248.s002.tif]
